# Neutrophil-derived reactive oxygen species promote tumor colonization

**DOI:** 10.1038/s42003-021-02376-8

**Published:** 2021-07-13

**Authors:** Jianghong Zhong, Qijing Li, Huqiao Luo, Rikard Holmdahl

**Affiliations:** 1 Medical Inflammation Research, Department of Medical Biochemistry and Biophysics, Karolinska Institute, Stockholm, Sweden; 2 Beijing Advanced Innovation Center for Big Data-Based Precision Medicine, Beihang University, Beijing, China; 3 Department of Hematology, the First Affiliated Hospital of Xi’an Jiaotong University, Xi’an, China; 4 The Second Affiliated Hospital of Xi’an Jiaotong University (Xibei Hospital), Xi’an, China

**Keywords:** Melanoma, Disease genetics, Neutrophils

## Abstract

A single-nucleotide polymorphism of neutrophil cytosolic factor 1 (*Ncf1*), leading to an impaired generation of reactive oxygen species (ROS), is a causative genetic factor for autoimmune disease. To study a possible tumor protection effect by the *Ncf1* mutation in a manner dependent on cell types, we used experimental mouse models of lung colonization assay by B16F10 melanoma cells. We observed fewer tumor foci in *Ncf1* mutant mice, irrespective of αβT, γδT, B-cell deficiencies, or of a functional *Ncf1* expression in CD68-positive monocytes/macrophages. The susceptibility to tumor colonization was restored by the human S100A8 (MRP8) promoter directing a functional *Ncf1* expression to granulocytes. This effect was associated with an increase of both ROS and interleukin 1 beta (IL-1β) production from lung neutrophils. Moreover, neutrophil depletion by anti-Ly6G antibodies increased tumor colonization in wild type but failed in the *Ncf1* mutant mice. In conclusion, tumor colonization is counteracted by ROS-activated and IL-1β-secreting tissue neutrophils.

## Abstract (web summary)

Zhong et al. investigate how mutations in the NOX2 subunit Ncf1 lead to reduced ROS formation and affect B16F10 lung metastasis. The study highlights neutrophils as key to detrimental ROS-formation and suggest the involvement of IL-1 beta and RAGE.

## Introduction

Immunotherapy has proven successful to reduce the cancer mortality in metastatic melanoma^1^, although the 5-year survival rates of patients with advanced melanoma are usually still <30% (ref. ^2^). It highlights the importance to study the role of earlier steps in immune responses to tumor colonization.

A recent finding is the identification of a single-nucleotide polymorphism in the *Ncf1* gene to be a genetic basis for autoimmunity^3–5^. The *Ncf1* gene encodes neutrophil cytosolic factor 1 (NCF1, earlier denoted p47^PHOX^), a cytosolic component of the NADPH oxidase 2 (NOX2) complex^6^. NCF1 is required for the NOX2 complex to trigger oxidative burst, i.e., an induced production of reactive oxygen species (ROS)^7^, and identified *Ncf1* mutations result in a decrease of ROS production^8,9^. NCF1 is highly expressed in neutrophils and CD68-positive cells^10^. CD68-expressing cells, predominantly macrophages, are a critical mediator regarding autoimmune disease susceptibility, exhibiting the properties of both innate and adaptive immune responses. The downstream effect of the *Ncf1* mutation includes regulation of T cells in autoimmune arthritis^10,11^ and encephalomyelitis^12^, exaggerated type I interferon signaling in lupus autoimmunity^13,14^, innate interleukin 17 (IL-17) induction in psoriatic diseases^15–17^, and impairment of the capacity to form neutrophil extracellular traps in patients with gout^18^ and gallstones^19^.

The induced immune responses by *Ncf1* mutations may on the other hand prevent cancer. It has been shown that myeloid cells producing ROS could suppress the cytotoxic T-cell response to tumor cells^20^. We and others have found that impaired NCF1 and NOX2 function are protective against the melanoma development^21,22^. We have now chosen the shared melanoma model B16F10 cell line, supplied by ATCC, to probe the mechanisms of cell-specific *Ncf1* mutations in tumor colonization.

In this study, we transferred the *Ncf1* mutation (Ncf1^*/*^) into mouse strains with different major histocompatibility complex (MHC) haplotypes on the *C57BL* genetic backgrounds. Using the MHC congenic mice in the mouse melanoma model, we observed a reduced number of lung colonies in *Ncf1* mutant mice. We excluded a critical role of T cells, B cells, and macrophages, but found that neutrophils were the most important cell for the *Ncf1* mutation-mediated protection. MRP8-restricted expression of a functional *Ncf1* restored the release of IL-1β from neutrophils and the susceptivity to tumor colonization. Thus, this study provides insights into the role of NOX2-derived ROS in driving tumor colonization through a neutrophil and IL-1β-dependent pathway.

## Results

### Protection despite the deficiencies of T and B cells

**Fig. 1:**
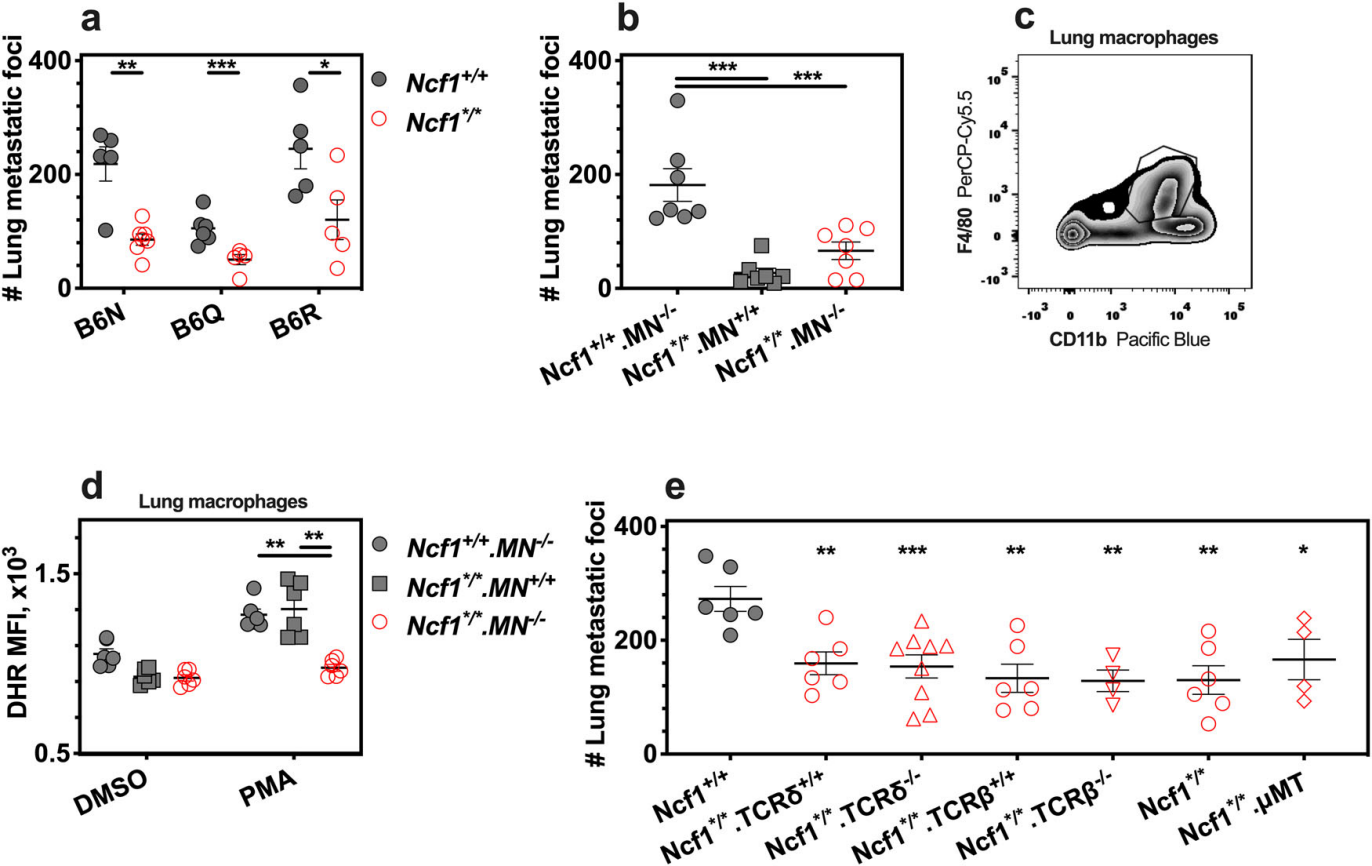
*Ncf1* mutation reduces lung colonies of B16F10 cells. The lungs were harvested from mice at day 10 after intravenous injection of B16F10 cells. **a** The number of tumor colonies in *Ncf1* mutant mice (Ncf1^*/*^) and wild-type controls (*Ncf1*^+/+^) of B6N.Ncf1^*/*^ (*n* = 7) versus *B6N.Ncf1*^+/+^ (*n* = 5), B6Q.Ncf1^*/*^ (*n* = 5) versus *B6Q.Ncf1*^+/+^ (*n* = 6), and B6R.Ncf1^*/*^ (*n* = 5) versus *B6R.Ncf1*^+/+^ (*n* = 5). **b** The number of tumor colonies in wild-type mice (*Ncf1*^+/+^.*MN*^*−/−*^, *n* = 7), MN mice (Ncf1^*/*^.*MN*^*+/+*^, *n* = 7), and *Ncf1* mutant mice (Ncf1^*/*^.*MN*^*−/−*^, *n* = 7). **c** A representative flow cytometry plot graph shows the gating of CD45^+^CD11b^+^F4/80^+^ cells (macrophages), and **d**, ROS production by DHR staining in flow cytometry, after lung cells were incubated ex vivo with PMA and DMSO as the control (six mice per group). **e** The number of tumor colonies in mice of Ncf1^*/*^.*TCRδ*^*−/−*^ (*n* = 9) versus Ncf1^*/*^.*TCRδ*^+/+^ (*n* = 6), Ncf1^*/*^.*TCRβ*^*−/−*^ (*n* = 4) versus Ncf1^*/*^.*TCRβ*^+/+^ (*n* = 6), and Ncf1^*/*^ (*n* = 6) versus Ncf1^*/*^.*μMT* (*n* = 4), compared with that in wild-type mice (*n* = 6). All results are shown as mean ± SEM. Mann–Whitney *U* test, *p < 0.05, **p < 0.01, and ***p < 0.001.

To study the role of redox regulation of tumor colonization, we introgressed the *Ncf1* mutation in mice with the MHC b, q, and r haplotypes on the shared *C57BL* genome background. Lung tumor foci were counted at day 10 after intravenous inoculation of B16F10 cells. We found that *Ncf1* mutant mice (Ncf1^*/*^) were protected from metastatic colonization, regardless of the MHC haplotype (Fig. 1a), arguing against redox control of allogenic-mediated rejection.

To further study the cell-type-specific effect of ROS deficiency, we investigated the role of macrophages and monocytes. We used the transgenic MN mice (Ncf1^*/*^.*MN*^+/+^) expressing NCF1 restricted to CD68-positive cells, and a fluorophore dihydrorhodamine 123 (DHR) to measure the ROS production of individual cells upon stimulation with phorbol 12-myristate 13-acetate (PMA). Our results show that the lung tumor foci were not changed in MN mice versus Ncf1 mutant mice (Fig. 1b), although ROS production was restored in macrophages/monocytes (Fig. 1c, d). Furthermore, *Ncf1*-mutated mice with deficiencies of T-cell receptor (TCR)-beta (Ncf1^*/*^.*TCRβ*^−/−^), TCR-delta (Ncf1^*/*^.*TCRδ*^−/−^), and B cell (Ncf1^*/*^.*μMT*) showed comparable levels of tumor colonies, similar to their littermate controls (Fig. 1d). These observations does not exclude an influence, but exclude a critically redox regulatory role of macrophages/monocytes, T and B cells in supporting lung colonization.

### Susceptibility to colonization restored by neutrophils

**Fig. 2:**
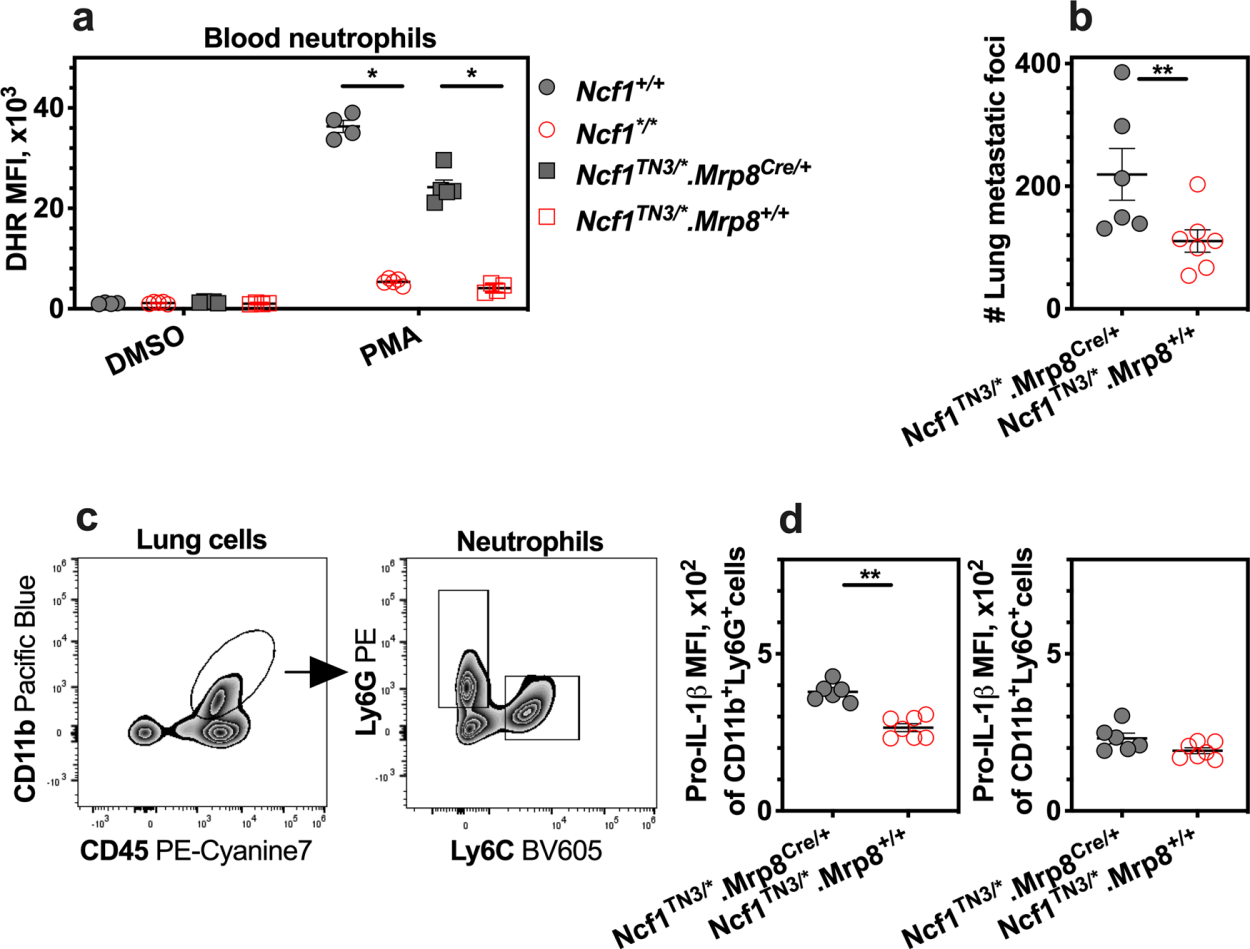
Tumor colonies are increased by neutrophil-restricted expression of functional *Ncf1*. **a** ROS production by neutrophil as assessed by flow cytometry with DHR, after blood cells were incubated ex vivo with PMA and DMSO as the control. The naive mice were used, including wild-type mice (*Ncf1*^+/+^, *n* = 4), *Ncf1* mutant mice (Ncf1^*/*^, *n* = 5), transgenic mice (*Ncf1*^TN3/*^.*Mrp8*^Cre/+^, *n* = 5), and their littermates (*Ncf1*^TN3/*^.*Mrp8*^+/+^, *n* = 4). **b** The number of tumor colonies on lungs per mouse was counted in transgenic mice and *Ncf1* mutant mice. **c** Representative flow cytometry plot graphs of CD45, CD11b, Ly6G, and Ly6C in lung cells. **d** The mean fluorescence intensity (MFI) of pro-IL-1β staining as the level of pro-IL-1β in the lung neutrophil was assessed by flow cytometry. In **b**, **c**, and **d**, the lungs were harvested from transgenic mice (*Ncf1*^TN3/*^.*Mrp8*^Cre/+^, *n* = 6) and *Ncf1* mutant mice (*Ncf1*^TN3/*^.*Mrp8*^+/+^, *n* = 7) to be analyzed at day 10 after intravenous injection of B16F10 cells. All results are shown as mean ± SEM. Mann–Whitney *U* test, *p < 0.05, **p < 0.01.

In the continuous search for the critical cells in mediating the *Ncf1* regulatory role, we investigated the role of neutrophils. We designed a conditional knock-in mouse model expressing a functional copy of the *Ncf1* gene in neutrophils by a Cre under control of the MRP8 promoter on *Ncf1*-deficient background (*Ncf1*^TN3/*^.*Mrp8*^Cre/+^). In prior to experiments, we validated the conditional expression of NCF1 in bone marrow neutrophils sorted from naive *Ncf1*^TN3/*^.*Mrp8*^Cre/+^ mice (Fig. S1a). We observed oxidative burst of blood neutrophils in the naive *Ncf1*^TN3/*^.*Mrp8*^Cre/+^ (Fig. 2a), whereas the ROS production of blood monocytes was not restored (Fig. S1b). We performed the tumor colonization assay with the knock-in mice. A higher number of tumor colonies were found in the lungs at day 10 of *Ncf1*^TN3/*^.*Mrp8*^Cre/+^ mice (Fig. 2b).

Previously, we and others have found a role of ROS in the regulation of IL-1β expression^12,23^. In this study, we show that ROS-sufficient neutrophils express an enhanced IL-1β in conditional knock-in mice (Fig. 2c, d). Consistently, expression of IL-1β was enhanced in lung neutrophils from wild-type mice (*Ncf1*^+/+^; Fig. S1c), as well as CD54 known as a molecular bridge^24^ at the interaction between tumor cells and neutrophils (Fig. S1d). These results suggest that neutrophils play a critical role to promote lung colonization by ROS through activating IL-1β secretion.

### Downstream mediators through IL-1β signals

**Fig. 3:**
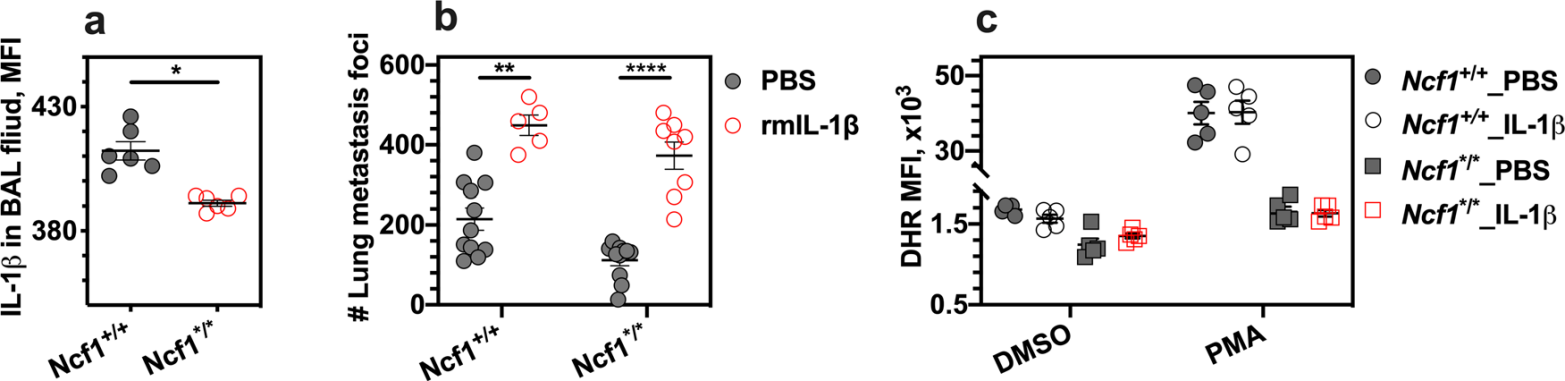
Administration of rmIL-1β increases lung colonization in *Ncf1* mutant mice. The bronchoalveolar lavage (BAL) fluids and lungs were harvested from wild-type mice (*Ncf1*^+/+^) and *Ncf1* mutant mice (Ncf1^*/*^) at day 10 after intravenous injection of B16F10 cells. **a** The level of IL-1β in the BAL fluid was assessed by flow cytometry. **b** The number of tumor colonies in lungs per mouse was counted in wild-type mice with injections of rmIL-1β (*n* = 5) versus PBS (*n* = 11), and *Ncf1* mutant mice with injections of rmIL-1β (*n* = 8) versus PBS (*n* = 11). **c** ROS production by neutrophils in the lungs was assessed by flow cytometry with DHR, after these cells were incubated ex vivo with PMA and DMSO as the control. The lung tissues were collected from wild-type mice with injections of rmIL-1β (*n* = 5) versus PBS (*n* = 5), and NCF1-deficient mice with injections of rmIL-1β (*n* = 5) versus PBS (*n* = 5) at day 10 post-injection of B16F10 cells. All results are shown as mean ± SEM. Mann–Whitney *U* test, *p < 0.05, **p < 0.01 and ***p < 0.001.

We next studied the effects of IL-1β in the tumor colonization model. For this purpose, we collected bronchoalveolar lavage fluid (BALF) at day 10 after intravenous injection of B16F10 cells. The analysis of flow cytometry data shows a decreased level of IL-1β in BALF from *Ncf1* mutant mice (Ncf1^*/*^; Fig. 3a). In addition, we observed the cytokine expressions of TNFα, IFN-γ, IL-4, and IL-1α in the same BALF, but there was no significant difference between *Ncf1* mutant group and wild-type controls (Fig. S1e).

To address a possible functional role, we treated the mice with recombinant mouse IL-1β (rmIL-1β) half an hour prior to the inoculation of B16F10 cells. We show that IL-1β treatment enhanced lung colonization, independently of the *Ncf1* polymorphism (Fig. 3b). The oxidative burst capability of lung neutrophils was also not affected by injection of rmIL-1β (Fig. 3c). Interestingly, we observed a reduced number of mature natural killer (NK) cells (CD45^+^CD3^−^NK1.1^+^) in lungs (Fig. S2a), i.e., CD27^−^KLRG1^+^ NK cells in an *Ncf1*-dependent manner, after a single injection of rmIL-1β in vivo (Fig. S2b, c). Mature NK cells can have a major role in controlling B16F10 lung colonization^25^, and then we studied the effect of NK cells by injecting NK1.1 antibody^26–28^. As expected, depletion of NK cells leads to an increase of the lung colonization of B16F10 tumor cells (Fig. S2d). However, the *Ncf1* mutation-mediated protection was consistent regardless of NK cell depletion (Fig. S2d). These results suggest that IL-1β promote tumor colonization as a downstream effect of ROS. NK cells may play a role in IL-1β signaling, but not critically in regulating the *Ncf1* effect.

### Enhanced colonization after neutrophil depletion

**Fig. 4:**
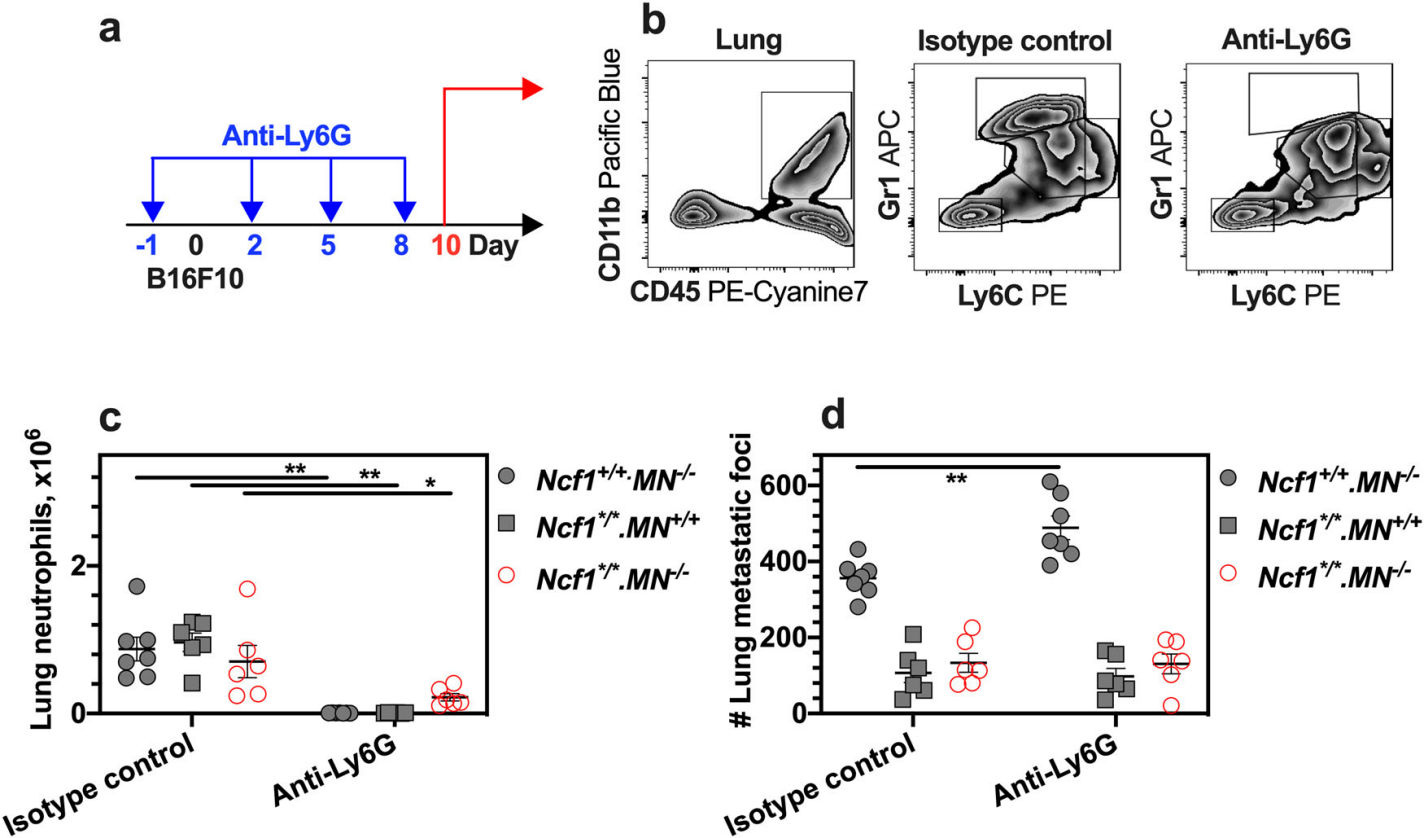
Treatment with anti-Ly6G antibodies increases lung colonization for mice with NCF1-expressing neutrophils. The studies used wild-type mice (*Ncf1*^+/+^.*MN*^*−/−*^), MN mice (Ncf1^*/*^.*MN*^*+/+*^), and *Ncf1* mutant mice (Ncf1^*/*^.*MN*^*–/−*^). B16F10 cells that were injected into mice at day 0, and the number of tumor colonies in lungs per mouse was counted at day 10 post-injection of B16F10 cells. **a** Administrations of anti-Ly6G antibodies were applied at days −1, 2, 5, and 8 post-injection of B16F10 cells. The study includes the wild-type mice with injections of anti-Ly6G antibody (clone:1A8; *n* = 7) versus isotype control (clone:2A3; *n* = 7), MN mice with injections of 1A8 (*n* = 6) versus 2A3 (*n* = 6), and *Ncf1* mutant mice with injections of 1A8 (*n* = 6) versus 2A3 (*n* = 6). **b** Representative flow cytometry plot graphs of CD11b, Gr1, and Ly6C in lung cells. **c** The number of neutrophils in lungs per mouse was counted. **d** The number of tumor colonies in lungs per mouse was counted in mice with injections of anti-Ly6G antibodies versus isotype control. All results are shown as mean ± SEM. Mann–Whitney *U* test, *p < 0.05, **p < 0.01.

Our data indicate that neutrophils play a critical role in ROS-mediated tumor colonization. To deplete neutrophils, we injected anti-Ly6G antibodies (clone 1A8) in wild-type mice, MN mice, and *Ncf1* mutant mice, and evaluated neutrophil infiltration at day 10 in the lungs of B16F10-exposed mice (Fig. 4a). The numbers of lung neutrophils (CD45^+^CD11b^+^Gr1^+^Ly6C^mid^) were reduced in all strains (Fig. 4b, c), while an increased number of monocytes (CD45^+^CD11b^+^Gr1^mid^Ly6C^hi^) was observed in wild-type mice (Fig. S3a). Contrary to the previous results that neutrophils depletion with anti-GR1 antibody (clone RB6-8C5) reduced the tumor growth^29,30^, and that 1A8 antibody treatment had no impact on lung colonization in *C57BL/6* mice^31^, our data shows that 1A8 antibodies enhanced lung colonization in *Ncf1* wild-type mice on the *C57BL/6* genetic background. Importantly however, this phenomenon was Ncf1 dependent as it was not seen in either *Ncf1* mutant or MN mice (Fig. 4d). A possible contribution to an increase of tumor colonies could be the expression of the receptor for advanced glycation endproducts (RAGE) on monocytes from wild-type mice (Fig. S3b). RAGE is known to be a receptor of MRP8 at the interaction between tumor cells and myeloid cells^24,32^. To determine the role of RAGE in oxidative regulation, we treated mice with the RAGE blocker FPS-ZM1 (ref. ^33^). Interesting, FPS-ZM1 reduced lung colonization in wild-type mice to the same low level as in *Ncf1* mutant mice (Fig. S3c, d). These results suggest that MRP8-RAGE signaling could activate monocytes to support tumor colonization after the ROS-sufficient neutrophil depletion.

## Discussion

A natural mutation in the *Ncf1* gene leading to an impaired production of ROS by the NOX2 complex protects against B16 melanoma tumor colonization of the lungs. The protection was reversed by conditional expression of a functional *Ncf1* gene in neutrophils. Subsequently, the neutrophil-derived IL-1β was demonstrated to promote lung colonization of B16F10 cells.

ROS could be produced by both tumor cells and infiltrating immune cells during metastatic colonization, and the location of ROS may determine oxidative effect on tumor growth. A recent study provided evidence that metabolic ROS support the metastasis process of tumor cells in clusters, compared with single cells^34^. The cluster could also be formatted between tumor cells and neutrophils. A phenomenon was reported that neutrophil–tumor clusters exhibit the capability to expand the metastatic potential of B16F10 cells^24^, but the role of ROS was not addressed. Normally, the NCF1–NOX2 complex is efficiently activated, dominating to the production of ROS in neutrophils. In this study, we validated with mouse models for the first time that a copy of functional *Ncf1* gene expression in neutrophils driven by the MRP8 promoter was enough to increase the level of lung metastatic foci.

ROS may shape the direct interaction between the injected tumor cells and infiltrating neutrophils. B16F10 cells lack detectable expression of ICAM-1 (ref. ^35^) and IL-1β^36^, but these two proteins could play pro-tumorigenic roles^24,37,38^. ICAM-1 expressed on neutrophils is important for neutrophil–tumor cell cluster formation^24^. ICAM-1 is absent or expressed at very low levels on circulating neutrophils, but could be induced by selected triggers^39^. In this study, we show that tumor-associated neutrophils expressed ICAM-1 and IL-1β in the lungs. *Ncf1* mutant mice exhibit a few number of ICAM-1-positive neutrophils. Importantly, the level of IL-1β expression was upregulated in the tissue neutrophils by conditional knock-in of a functional *Ncf1* gene. A single injection of IL-1β compensated for the effect of ROS deficiency on tumor colonization, increasing tumor colonies to a high level. Recently, it has been reported that deficiency of the developmental endothelial locus–1 in the host may promote neutrophil accumulation in the lung metastatic niche, where neutrophil-released ROS mediates a direct interaction with B16F10 cells^40^.

Another direct interaction between neutrophils and tumor cells could involve the RAGE receptor on B16F10 cells, of which the MRP8 is a potent ligand^41^. The myeloid cells, including neutrophils, monocytes, and the interstitial macrophage-like phenotype, might express both the NCF1–NOX2 complex and MRP8, referred to the formation of the pre-metastatic microenvironment that is required for blood tumor cells to engraft at the lung niche^42^. Thus, it is difficult to distinguish the neutrophils from monocytes/macrophages on the immunoregulation. Expression of a functional *Ncf1* gene in CD68-positive cells can reduce the pathogenicity of T cells in experimental autoimmune arthritis models^10^. In this study, we show that the *Ncf1* mutation protected from tumor colonization, irrespective of the CD68-expressing cell status. What is more, depletion of ROS-sufficient neutrophils with 1A8 antibodies was likely to trigger the ROS-dependent RAGE expression on Ly6C monocytes.

In addition, NK cells could play a prominent role in the protection against lung colonization. Aydin and colleagues studied the role of NK cells in *Nox2*-knockout mouse models by anti-NK1.1 antibody injection, finding an NK cell-associated anti-metastatic effect of histamine dihydrochloride in *Nox2* wild-type mice, but not in *Nox2*-knockout mice^26,27^. Li and colleagues investigated the role of the cytokine G-CSF in tumor models on use of both NOD-*scid* and NSG mice, suggesting a neutrophil-mediated role of NK cells in lung metastasis^28^. It is known that innate lymphoid cells are also deficient in NSG mice^43^. To study the importance of neutrophils, Boivin and colleagues found that neutrophils could even expand in blood and spleens after anti-Ly6G treatments at a low dose^29^. Clearly, NK cells play an important role in the protection of lung colonization, but it remains unknown whether NK cells are involved with the protective effect of the NCF1–NOX2 complex. It should be pointed out that the small compound histamine dihydrochloride might not specifically affect NK cells, and there is also genetic difference between the knockout mouse strains and naturally mutant strains. For an example, both *Nox2*-knockout mice and *Ncf1*-knockout mice have been reported to be protected from experimental autoimmune encephalomyelitis^44^, but the *Ncf1* mutant mice exhibited an enhanced autoimmune encephalomyelitis^12,45^. Therefore, we further studied the role of NK cells in the *Ncf1* mutant mice using anti-NK1.1 antibody^26^. However, we found that NK cell depletion, by anti-NK1.1 treatments, enhanced tumor metastasis independent of the *Ncf1* mutation.

In summary, we used naturally mutant mouse models of the *Ncf1* gene to screen the cell-type-specific effect of the NOX2 complex deficiency, and then found out that only *Ncf1* expression in neutrophils can restore the susceptibility to tumor colonization. We found that the *Ncf1* competent neutrophils, exhibiting the capability of functional ROS induction and IL-1β signaling, can promote lung colonization. As the *Ncf1* expression is polymorphic in both humans and experimental animals, and since redox regulation is a key pathophysiologic process, this observation could be of key importance not only to understand the basis of tumor metastasis, but also to improve cancer treatment.

## Methods

### Animals

Founders of *B6N* (*C57BL/6N*) and *B10RIII* mice are originally from the JAX Lab (Bar Harbor, Maine), and the MHC congenic *B6Q* (*C57/B6N.Q/rhd*) and *B10Q* (*C57/B10N.Q/rhd*) mice have been fully backcrossed and maintained by the Holmdahl laboratory as inbred lines (rhd). The congenic mice of *B6R* strain have been established by an initial cross of a *B10RIII* mouse with *B6N* mice, followed by at least eight times of repeated backcrossing to *B6N* mice. A mutation in the *Ncf1* gene (m1j) in the *B6N* mice, designated as *B6N.Ncf1*^m1j/m1j^ (B6N.Ncf1^*/*^) impairs the expression of the *Ncf1* gene, thereby totally blocking the function of the NOX2 complex. The derived *Ncf1* mutant mouse strains include B6Q.Ncf1^*/*^, B10Q.Ncf1^*/*^, and B6R.Ncf1^*/*^. The transgenic MN mice with the human CD68 promoter exhibit macrophage/monocytes-restricted NCF1 expression (B10Q.Ncf1^*/*^.*MN*^+/+^)^10^. The MRP8-Cre transgenic mice (stock no: 021614) were used to cross with the targeted *Ncf1* mutant mice (*B6Q.Ncf1*^TN3/TN3^), producing the mice with a conditional knock-in of a functional *Ncf1* gene in neutrophils, i.e., *B6Q.Ncf1*^TN3/*^.*Mrp8*^Cre/+^. TCRβ-deficient mice (stock no: 002118, *B6.129P2-Tcrb*^tm1Mom^/J), TCRδ-deficient mice (stock no: 002120, *B6.129P2-Tcrd* ^tm1Mom^/J), B-cell-deficient mice (stock no: 002288, *B6.129S2-Ighm* ^tm1Cgn^/J) were obtained from The Jackson Laboratory and were fully backcrossed to our B10Q.Ncf1^*/*^ mice to get B10Q.Ncf1^*/*^.*TCRβ*^−/−^ mice, B10Q.Ncf1^*/*^.*TCRδ*^−/−^ mice, B10Q.Ncf1^*/*^.*μMT* mice, respectively. The primers for *TCRβ* genotyping are as following: 5′-GCT ACT TCC ATT TGT CAC GTC C-3′ (mutant forward), 5′-CCC CAC CCA GTA TAG GAC AG-3′ (wild-type forward), 5′-CCT CAA CCC AGA ATG ATC TTG-3′ (common). The primers for *TCRδ* genotyping are as following: 5′-CTT GGG TGG AGA GGC TAT TC-3′ (mutant forward), 5′-AGG TGA GAT GAC AGG AGA TC-3′ (mutant reverse), 5′-CAA ATG TTG CTT GTC TGG TG-3′ (wild-type forward), 5′-GTC AGT CGA GTG CAC AGT TT-3′ (wild-type reverse). The primers for *μMT* genotyping as following: 5′-CCG TCT AGC TTG AGC TAT TAG G-3′ (common), 5′-GAA GAG GAC GAT GAA GGT GG-3′ (wild type), 5′-TTG TGC CCA GTC ATA GCC GAA T-3′ (mutant reverse). The primers for *Cre* genotyping are as following: 5′-GCG GTC TGG CAG TAA AAA CTA TC-3′ (transgene forward), 5′-GTG AAA CAG CAT TGC TGT CAC TT-3′ (transgene reverse), 5′-CTA GGC CAC AGA ATT GAA AGA TCT-3′ (internal positive control forward), 5′-GTA GGT GGA AAT TCT AGC ATC ATC C-3′ (internal positive control reverse). Littermate male mice were used in our experiments, and the identity was blinded for the investigator. Mice were housed under specific pathogen-free conditions in individually ventilated cages with wood shaving bedding in a climate-controlled environment having a 12-h light/dark cycle. We have mixed experimental cages of 6- to 8-week-old homozygous littermates. Each adult mouse weighed ~25 g. Experimental groups were randomized and distributed among mixed cages. The animal study protocols were approved by the Stockholm regional animal ethics committee, Sweden (N288/15).

### Antibodies

The following antibodies were purchased from BioLegend, as CD45 (clone: 30-F11, APC or PE-Cyanine7), CD11b (clone: M1/70, Pacific Blue or APC), Ly6G (clone: 1A8, PerCP/Cy5.5), Ly6C (clone: HK1.4, APC or FITC), F4/80 (clone: BM8, PerCP/Cy5.5 or FITC), CD11c (clone: N418, APC or PE), and CD54 (clone: YN1/1.7.4, PE or FITC). Antibodies for CD16/CD32 (clone: 2.4G2, purified), Gr1 (clone: RB6-8C5, APC), CD115 (clone: T38-320, PE), CD3ε (clone: 145-2C11, FITC), NK1.1 (clone: PK136, Pacific Blue), CD27 (clone: LG.3A10, PerCP/Cy5.5), KLRG1 (clone: 2F1, APC), CD107a (clone: 1D4B, PE), and IFN-γ (clone: 1D4B, PE) were purchased from BD Biosciences. Antibodies for IL-1β (clone: NJTEN, FITC) were purchased from eBioscience. Antibodies for NCF1 (clone: D-10, FITC) were purchased from Santa Cruz Biotechnology. Antibodies for RAGE (clone: 697023) were purchased from R&D Systems. The usage of antibodies is according to the suggestions from the source companies, and the classical dilution ratio of the stock solution is 1:200 for flow cytometry staining.

### Western blot

Bone marrow cells were flushed from one femur and one tibia per mouse with RPMI media (ThermoFisher, catalog no. 61870044) supplemented by 10% FBS. Red blood cells were lysed with ACK (Ammonium-Chloride-Potassium) buffer^46^. Neutrophils were magnetically isolated using anti-Ly6G antibody (Clone: 1A8, BioLegend) according to manufacturer’s instructions from Miltenyi Biotec. Purity was checked by Attune™ NxT Flow Cytometer. The cells were lysed with RIPA Buffer (Pierce, Thermo Scientific), and then subjected to electrophoresis and transferred to nitrocellulose membranes (ThermoFisher, catalog no. LC2001, Sweden). Membranes were incubated with antibodies for NCF1 (Clone: D-10, Santa Cruz Biotechnology) and cyclophilin A (ThermoFisher, catalog no. PA1-025, Sweden) at 1:1000 dilution, followed by incubation with HRP-conjugated goat anti-rabbit IgG (ThermoFisher, catalog no. 31460) at 1:20,000 dilution. The ChemiDoc XRS + Gel imaging system (Bio-Rad Laboratories AB, Sweden) was used for chemiluminescence detection.

### Cytometric beads array

Cytokine levels in BALF were measured by flow cytometry using BD Cytometric Bead Array Mouse/Rat Soluble protein Master Buffer kit (IL-1α, IL-1β, IL-4, TNF-α, and IFN-γ), according to the manufacturer’s instruction (BD Biosciences, US).

### Tumor transplantation

For studies of lung colonization, each mouse was intravenously injected with 1.5 × 10^5^ B16F10 cells (ATCC CRL-6475) dissolved in 100 μL of phosphate-buffered saline (PBS, 1×; Life Technologies Europe BV, Sweden) on day 0. Mice were sacrificed to harvest lung tissues at the indicated time. Tumor colonies of B16F10 cells on the surface of lung lobes per mouse were counted under Leica M60 stereo zoom microscope (Leica, Micromedic AB, Sweden).

### Administration of recombinant mouse IL-1β protein

Half an hour prior to the inoculation of tumor cells, the experimental mouse was administrated intraperitoneally with a single injection of 1 μg rmIL-1β protein (BioLegend, Catalog Number 575106, Sweden) dissolved in 200 μL of PBS. The controls were injected with 200 μL of PBS.

### Ly6G ligation

Neutrophils were depleted by intraperitoneal injections of 250 μg anti-Ly6G antibody (BioXCell, Clone 1A8) every 3 days, and rat IgG2a isotype control (BioXCell, Clone 2A3) were used in the same way. The exact methods of injections were to inject at day −1, 2, 5, and 8 post-injection of B16F10 cells.

### Administration of RAGE blocker FPS-ZM1

Mice were administrated intraperitoneally with 25 μg FPS-ZM1 (Merck, Catalog Number 553030, Sweden) dissolved in 200 μL of PBS per time. PBS was used as a control. Mice received FPS-ZM1 treatments on days −1, 0, 1, and 2 after intravenous injection of B16F10 cells.

### Flow cytometry analysis

The single-cell suspension derived from blood and lung tissues was analyzed with flow cytometry. To prepare the single-cell suspensions, the lungs were dissected into smaller fragments and digested in PBS with 2 mg/mL collagenase type IV (Sigma, C5138, CAS no. 9001-12-1, Sweden), 100 U/mL DNase I (Roche, Sigma, Catalog Number 04716728001, Sweden), and 2 mM EDTA (Sigma, CAS no. 60-00-4, Sweden). The cell density was counted by using Sysmex KX-21N automated hematology analyzer (Sysmex Corporation, NY). The cell samples were stained with a LIVE/DEAD® fixable near-IR dead cell stain (ThermoFisher, catalog no. L10119, Sweden). After an anti-mouse CD16/CD32 Fc block, extracellular antigens were stained 20 min at 4 °C in PBS with 1% fetal bovine serum (FBS, Gibco, ThermoFisher, catalog no. 26140079, USA). To measure intracellular ROS, the staining of 3 μM DHR (ThermoFisher, Catalog Number D23806, Sweden) was conducted, respectively, after cell surface markers staining, followed by stimulation of 100 ng/mL of PMA (Sigma-Aldrich Co., CAS No. 16561-29-8, Sweden) for 30 min. To detect the intracellular expression of cytokines, the cells were stimulated with 100 ng/mL of PMA and 1 μg/mL of ionomycin in the presence of 5 μg/mL of brefeldin A (BFA) for 4 h at a humidified 37 °C, 5% CO_2_ incubator. The stock solutions of PMA, ionomycin (ThermoFisher, Catalog Number I24222, Sweden), and BFA (ThermoFisher, catalog no. B7450, Sweden) were prepared with dimethylsulfoxide (DMSO, Sigma-Aldrich Co., CAS no. 67-68-5, Sweden). For intracellular cytokine staining, cells were fixed and permeabilized using BD cytofix/cytoperm solution (BD Biosciences, catalog no. 554714, Sweden). Samples were acquired using BD LSR II flow cytometer. The workstation is managed by FACSDiva software version 8.0 (BD Biosciences), and the data were analyzed using the FlowJo software version 10.5.3 (TreeStar, Inc., OR).

### Statistics and reproducibility

Statistical analyses were performed with Graph Prism software, version 8.2.1 (GraphPad Software, San Diego, USA). Unless otherwise stated, the Mann–Whitney *U* test was used to compare the means of two groups. All results are shown as mean ± standard error of the mean. *P* value < 0.05 was considered as significant: *p < 0.05, **p < 0.01, ***p < 0.001, and ****p < 0.0001.

### Reporting summary

Further information on research design is available in the Nature Research Reporting Summary linked to this article.

## Supplementary information

Supplementary Information

Description of Additional Supplementary Files

Supplementary Data 1

Reporting Summary

## Data Availability

Study data are included in the article and the Supplementary Materials. No datasets were generated or analyzed during the current study.
