# Supplementary material for: Neutrophil-derived reactive oxygen species promote tumor colonization
Source: Commun Biol. 2021 Jul 13;4:865. doi: 10.1038/s42003-021-02376-8 (PMC8277858; doi:10.1038/s42003-021-02376-8)
Supplement: Supplementary file 1 — Supplementary Information [file 42003_2021_2376_MOESM1_ESM.pdf]

Neutrophil-derived reactive oxygen species promote tumor colonization

Jianghong Zhong[1,2,*], Qijing Li[3,1], Huqiao Luo[1], Rikard Holmdahl[1,4,*]

[1]Medical Inflammation Research, Department of Medical Biochemistry and Biophysics, Karolinska Institute, Stockholm 17177, Sweden

[2]Beijing Advanced Innovation Center for Big Data-Based Precision Medicine, Beihang University, Beijing 100083, China

[3]Department of Hematology, the First Affiliated Hospital of Xi'an Jiaotong University, Xi'an 710061, China

[4]The Second Affiliated Hospital of Xi'an Jiaotong University (Xibei Hospital), Xi'an 710004, China

*** Correspondence**: jzhong@buaa.edu.cn (J.Z.), rikard.holmdahl@ki.se (R.H.)

ORCIDs

Jianghong Zhong: https://orcid.org/0000-0002-1757-6642

Rikard Holmdahl: https://orcid.org/0000-0002-4969-2576

Supplementary Information

Figure S1, S2, S3

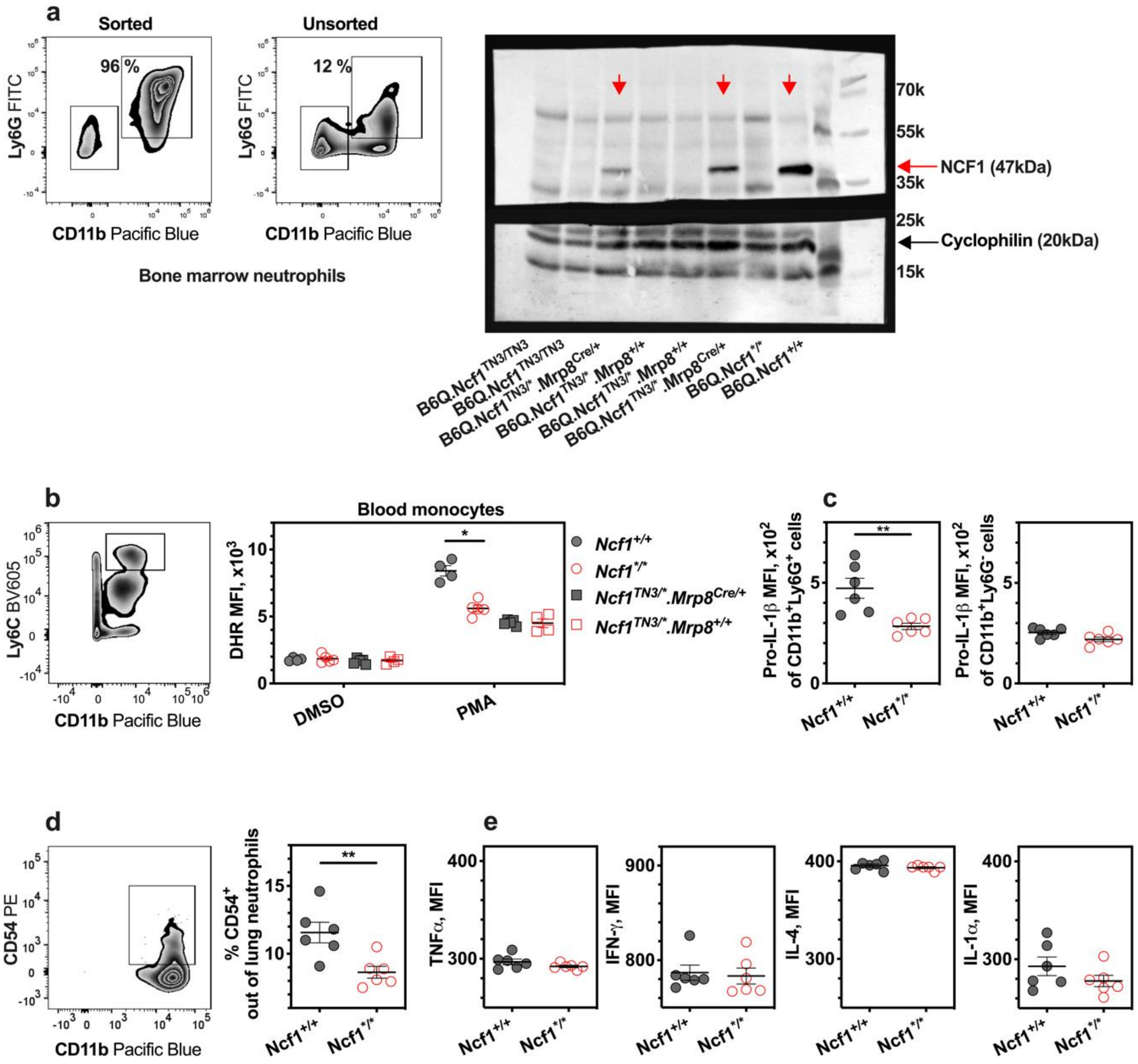

Figure S1. Expression and function of NCF1 and IL-1β in neutrophils. **a**, Representative flow cytometry plot graphs of sorted neutrophils from naïve mice, and their NCF1 expression. The shown samples were collected from transgenic mice (*Ncf1*$^{\text{TN3/*}}$.*Mrp8*$^{\text{Cre/+}}$), *Ncf1* mutant mice (*Ncf1*$^{\text{TN3/TN3}}$, *Ncf1*$^{*/*}$, *Ncf1*$^{\text{TN3/*}}$.*Mrp8*$^{+/+}$) and wild type mice (*Ncf1*$^{+/+}$). The red arrows point to the bands of NCF1. **b**, The oxidative burst of blood monocytes was stained by a fluorescent dye DHR. The naïve mice were used, including wild type mice (*Ncf1*$^{+/+}$, n=4), *Ncf1* mutant mice (*Ncf1*$^{*/*}$, n=5), transgenic mice (*Ncf1*$^{\text{TN3/*}}$.*Mrp8*$^{\text{Cre/+}}$, n=5) and their littermates (*Ncf1*$^{\text{TN3/*}}$.*Mrp8*$^{+/+}$ n=4). **c**, Pro-IL-1β expression of lung neutrophils were shown in *Ncf1* mutant mice (*Ncf1*$^{*/*}$, n=6) and wild

type mice (*Ncf1*$^{+/+}$, n=6). **d**, A representative flow cytometry plot graph of CD11b and CD54, and then the frequencies of CD54 positive subsets in lung neutrophils. In **c** and **d**, the lungs were collected at day 10 after intravenous injection of B16F10 cells. **e**, the cytokine levels in the bronchoalveolar lavage (BAL) fluid were assessed by flow cytometry. In **c**, **d**, and **e**, The lungs and BAL fluids were harvested from wild type mice (*Ncf1*$^{+/+}$) and *Ncf1* mutant mice (*Ncf1*$^{*/*}$) at day 10 after intravenous injection of B16F10 cells. All results are shown as mean ± SEM. Mann-Whitney U test was conducted. **$p < 0.01$.

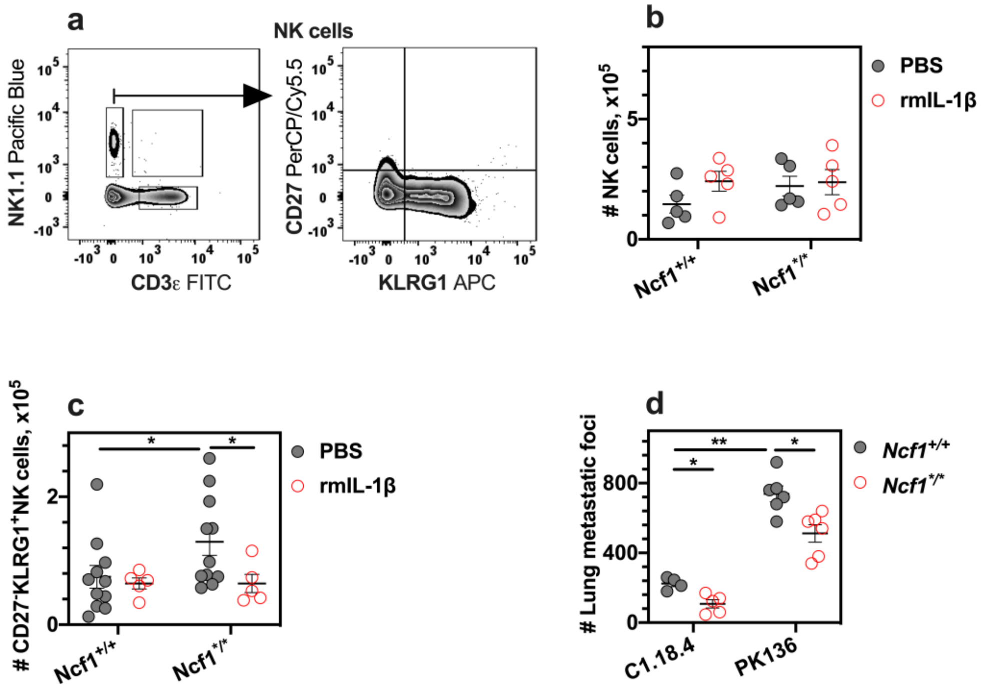

Figure S2. Flow cytometry analysis of lung natural killer (NK) cells is shown. The lungs were harvested at day 10 after intravenous injection of B16F10 cells. **a**, Representative flow cytometry plot graphs of NK1.1, CD3ε, CD27 and KLRG1 staining in lung cells. **b**, The number of NK cells in lungs per mouse was counted. **c**, The number of mature ($CD27^{-}KLRG1^{+}$) NK cells in lungs per mouse was counted. The lung tissues were collected from wild type mice (*Ncf1*$^{+/+}$) with injections of rmIL-1β (n=5) versus PBS (n=11), and NCF1 deficient mice (*Ncf1*$^{*/*}$) with injections of rmIL-1β (n=5) versus PBS (n=11). **d**, the number of tumor colonies in lungs per mouse was counted in mice with injections of anti-NK1.1 antibody (BioXCell, clone, PK136; 6 mice per group) versus isotype control (BioXCell, clone, C1.18.4; 5 mice per group), which were intraperitoneally injected with a dose of 250 μg per mouse at 4 days and 2 days before B16F10 cell inoculation. All results are shown as mean ± SEM. Mann-Whitney U test, *$p < 0.05$.

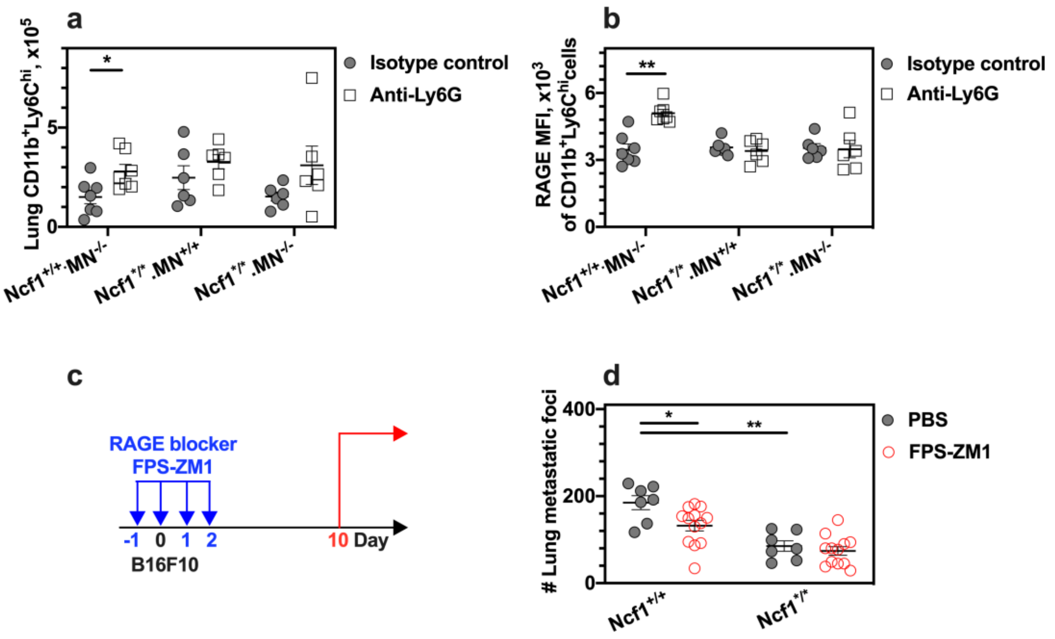

Figure S3. RAGE blocker decreases lung colonization in wild type mice. The studies used wild type mice ($Ncf1^{+/+}.MN^{-/-}$), MN mice ($Ncf1^{*/*}.MN^{+/+}$), and *Ncf1* mutant mice ($Ncf1^{*/*}.MN^{-/-}$). The lungs per mouse were analyzed at day 10 post-injection of B16F10 cells. **a**, The number of Ly6C^{hi} monocytes in the lungs per mouse is shown after anti-mouse Ly6G antibodies treatments, and **b**, the MFI of RAGE staining for Ly6C^{hi} monocytes. In **a** and **b**, The study includes the wild type mice with injections of 1A8 (n=7) versus 2A3 (n=7), MN mice with injections of 1A8 (n=6) versus 2A3 (n=6), and *Ncf1* mutant mice with injections of 1A8 (n=6) versus 2A3 (n=6). Administrations of anti-Ly6G antibodies were applied at day -1, 2, 5, and 8 post-injection of B16F10 cells. **c**, FPS-ZM1 was intraperitoneally injected into mice one day before injection of B16F10 cells, and at day 0, 1 and 2 after injection of tumors. **d**, Tumor colonies of lungs were counted. In **c** and **d**, the mice groups are made of wild type mice with injections of FPS-ZM1 (n=13) versus PBS (n=7), and *Ncf1* mutant mice with injections of FPS-ZM1 (n=12) versus PBS (n=7). All results are shown as mean ± SEM. Mann-Whitney U test, $*p < 0.05$, $**p < 0.01$, and $***p < 0.001$.
