# Supplementary material for: Neutrophil-derived reactive oxygen species promote tumor colonization
Source: Commun Biol. 2021 Jul 13;4:865. doi: 10.1038/s42003-021-02376-8 (PMC8277858; doi:10.1038/s42003-021-02376-8)
Supplement: Supplementary file 2 — Description of Additional Supplementary Files [file 42003_2021_2376_MOESM2_ESM.pdf]

Description of Additional Supplementary Files

File name: Supplementary Data 1

Description: Source data for all graphs and charts.
