# Supplementary material for: Neutrophil-derived reactive oxygen species promote tumor colonization
Source: Commun Biol. 2021 Jul 13;4:865. doi: 10.1038/s42003-021-02376-8 (PMC8277858; doi:10.1038/s42003-021-02376-8)
Supplement: Supplementary file 4 — Reporting Summary [file 42003_2021_2376_MOESM4_ESM.pdf]

nature research

Corresponding author(s): Jianghong Zhong; Rikard Holmdahl
COMMSBIO-21-0752C

Last updated by author(s): Jun 3, 2021

Reporting Summary

Nature Research wishes to improve the reproducibility of the work that we publish. This form provides structure for consistency and transparency in reporting. For further information on Nature Research policies, see our Editorial Policies and the Editorial Policy Checklist.

Statistics

For all statistical analyses, confirm that the following items are present in the figure legend, table legend, main text, or Methods section.

n/a	Confirmed	
☐	☒	The exact sample size (*n*) for each experimental group/condition, given as a discrete number and unit of measurement
☐	☒	A statement on whether measurements were taken from distinct samples or whether the same sample was measured repeatedly
☐	☒	The statistical test(s) used AND whether they are one- or two-sided<br>*Only common tests should be described solely by name; describe more complex techniques in the Methods section.*
☐	☒	A description of all covariates tested
☐	☒	A description of any assumptions or corrections, such as tests of normality and adjustment for multiple comparisons
☐	☒	A full description of the statistical parameters including central tendency (e.g. means) or other basic estimates (e.g. regression coefficient) AND variation (e.g. standard deviation) or associated estimates of uncertainty (e.g. confidence intervals)
☐	☒	For null hypothesis testing, the test statistic (e.g. F, t, r) with confidence intervals, effect sizes, degrees of freedom and P value noted<br>*Give P values as exact values whenever suitable.*
☐	☒	For Bayesian analysis, information on the choice of priors and Markov chain Monte Carlo settings
☐	☒	For hierarchical and complex designs, identification of the appropriate level for tests and full reporting of outcomes
☐	☒	Estimates of effect sizes (e.g. Cohen's *d*, Pearson's *r*), indicating how they were calculated

Our web collection on statistics for biologists contains articles on many of the points above.

Software and code

Policy information about availability of computer code

Data collection	FACSDiva software version 8.0 (BD Biosciences)
Data analysis	Graph Prism software, version 8.4.3 (GraphPad Software, San Diego, UAS); FlowJo software version 10.5.3 (TreeStar, Inc., OR)

For manuscripts utilizing custom algorithms or software that are central to the research but not yet described in published literature, software must be made available to editors and reviewers. We strongly encourage code deposition in a community repository (e.g. GitHub). See the Nature Research guidelines for submitting code & software for further information.

Data

Policy information about availability of data

All manuscripts must include a data availability statement. This statement should provide the following information, where applicable:
- Accession codes, unique identifiers, or web links for publicly available datasets
- A list of figures that have associated raw data
- A description of any restrictions on data availability

Authors can confirm that all relevant data are included in the paper and/ or its supplementary information files

Field-specific reporting

Life sciences study design

All studies must disclose on these points even when the disclosure is negative.

Sample size	The sample size and statistical method were determined in each figure legend.
Data exclusions	No data were excluded from analysis.
Replication	The replication was determined in each figure legend.
Randomization	Research participants were randomly assigned to the sample groups.
Blinding	The investigators were blinded to group allocation during data collection and analysis.

Reporting for specific materials, systems and methods

We require information from authors about some types of materials, experimental systems and methods used in many studies. Here, indicate whether each material, system or method listed is relevant to your study. If you are not sure if a list item applies to your research, read the appropriate section before selecting a response.

Materials & experimental systems

n/a	Involved in the study
☐	☒ Antibodies
☐	☒ Eukaryotic cell lines
☒	☐ Palaeontology and archaeology
☐	☒ Animals and other organisms
☒	☐ Human research participants
☒	☐ Clinical data
☒	☐ Dual use research of concern

Methods

n/a	Involved in the study
☒	☐ ChIP-seq
☐	☒ Flow cytometry
☒	☐ MRI-based neuroimaging

Antibodies

Antibodies used	The following antibodies were purchased from BioLegend, as CD45 (clone: 30-F11, APC or PE-Cyanine7), CD11b (clone: M1/70, Pacific Blue or APC), Ly6G (clone: 1A8, PerCP/Cy5.5), Ly-6C (clone: HK1.4, APC or FITC), F4/80 (clone: BM8, PerCP/Cy5.5 or FITC), CD11c (clone: N418, APC or PE), CD54 (clone: YN1/1.7.4, PE or FITC).<br>Antibodies for CD16/CD32 (clone: 2.4G2, purified), Gr-1 (clone: RB6-8C5, APC), CD115 (clone: T38-320, PE), CD3ε (clone: 145-2C11, FITC), NK1.1 (clone: PK136, Pacific Blue), CD27 (clone: LG.3A10, PerCP/Cy5.5), KLRG1 (clone: 2F1, APC), CD107a (clone: 1D4B, PE), IFN-γ (clone: 1D4B, PE) were purchased from BD Biosciences.<br>Antibodies for IL-1β (clone: NJTEN, FITC) were purchased from eBioscience.<br>Antibodies for NCF1 (clone: D-10, FITC) were purchased from Santa Cruz Biotechnology.
Validation	Each product datasheet has listed the antibody standard validation, with the most common examples of these being ELISA and flow cytometry on the manufacturer's website and relevant citations.

Eukaryotic cell lines

Policy information about cell lines

Cell line source(s)	Murine melanoma B16F10 cells (ATCC® CCL-6475™) were purchased from American Type Culture Collection (ATCC).
Authentication	The animal study protocols were approved by the Stockholm regional animal ethics committee, Sweden (N288/15).
Mycoplasma contamination	We confirmed that the B16F10 cell line was tested negative for mycoplasma contamination.
Commonly misidentified lines (See ICLAC register)	None.

Animals and other organisms

Policy information about studies involving animals; ARRIVE guidelines recommended for reporting animal research

Laboratory animals	Founders of B6N (C57BL/6N), and B10RIII mice are originally from the JAX Lab (Bar Harbor, Maine) and the MHC congenic B6Q (C57/B6N.Q/rhd) and B10Q (C57/B10N.Q/rhd) mice have been fully backcrossed and maintained by the Holmdahl laboratory as inbred lines (rhd). The congenic mice of B6R strain have been established by an initial cross of a B10RIII mouse with B6N mice, followed by at least eight times of repeated backcrossing to B6N mice. A mutation in the Ncf1 gene (m1j) in the B6N mice, designated as

B6N.Ncf1m1j/m1j (B6N.Ncf1*/*) impairs the expression of the Ncf1 gene, thereby totally blocking the function of the NOX2 complex. The derived Ncf1-mutant mouse strains include B6Q.Ncf1*/*, B10Q.Ncf1*/*. B6R.Ncf1*/*. The transgenic MN mice with the human CD68 promoter exhibit macrophage/monocytes-restricted NCF1 expression (B10Q.Ncf1*/*.MN+/+) 10. The MRP8-Cre transgenic mice (Stock No: 021614) were used to cross with the targeted Ncf1 mutant mice (B6Q.Ncf1TN3/TN3), producing the mice with a conditional knock-in of a functional Ncf1 gene in neutrophils, i.e., B6Q.Ncf1TN3/*.Mrp8Cre/+ . TCRβ-deficient mice (Stock No: 002118, B6.129P2-Tcrbtm1Mom/J), TCRδ-deficient mice (Stock No: 002120, B6.129P2-Tcrd tm1Mom/J), B-cell deficient mice (Stock No: 002288, B6.129S2-Ighm tm1Cgn/J) were obtained from The Jackson Laboratory and were fully backcrossed to our B10Q.Ncf1*/* mice to get B10Q.Ncf1*/*.TCRβ-/- mice, B10Q.Ncf1*/*.TCRδ-/- mice, B10Q.Ncf1*/*.µMT mice, respectively.

Wild animals	The study did not involve wild animals.
Field-collected samples	Mice were housed under specific pathogen-free conditions in individually ventilated cages with wood shaving bedding, a paper napkin as enrichment, and in a climate-controlled environment having a 12-hour light/dark cycle. All of the field-collected samples were under the standard environments.
Ethics oversight	The animal study protocols were approved by the Stockholm regional animal ethics committee, Sweden (N288/15).

Note that full information on the approval of the study protocol must also be provided in the manuscript.

Flow Cytometry

Plots

Confirm that:

- [x] The axis labels state the marker and fluorochrome used (e.g. CD4-FITC).
- [x] The axis scales are clearly visible. Include numbers along axes only for bottom left plot of group (a 'group' is an analysis of identical markers).
- [x] All plots are contour plots with outliers or pseudocolor plots.
- [x] A numerical value for number of cells or percentage (with statistics) is provided.

Methodology

Sample preparation	The single-cell suspensions derived from blood and lung tissues were analyzed with flow cytometry. To prepare the single-cell suspensions, the lungs were dissected into smaller fragments and digested in PBS with 2 mg/ml collagenase type IV (Sigma, C5138, CAS Number 9001-12-1, Sweden), 100 U/ml DNase I (Roche, Sigma, Catalog Number 04716728001, Sweden), and 2 mM EDTA (Sigma, CAS Number 60-00-4, Sweden).
Instrument	The cell density was counted by using Sysmex KX-21N automated hematology analyzer (Sysmex Corporation, NY). Samples were acquired using BD LSR II flow cytometer.
Software	The flow cytometer workstation is managed by FACSDiva software version 8.0 (BD Biosciences), and the data were analyzed using the FlowJo software version 10.5.3 (TreeStar, Inc., OR).
Cell population abundance	The single-cell suspensions derived from blood and lung tissues were directly analyzed with flow cytometry, and all the cell population frequencies were determined in figures and figure legends.
Gating strategy	The gating strategy has been shown in the figures and supplementary data.

- [x] Tick this box to confirm that a figure exemplifying the gating strategy is provided in the Supplementary Information.
